# Effects of postmastectomy radiotherapy on prognosis in different tumor stages of breast cancer patients with positive axillary lymph nodes

**DOI:** 10.7497/j.issn.2095-3941.2014.02.007

**Published:** 2014-06

**Authors:** Miao-Miao Jia, Zhi-Jie Liang, Qin Chen, Ying Zheng, Ling-Mei Li, Xu-Chen Cao

**Affiliations:** ^1^The First Department of Breast Cancer, Tianjin Medical University Cancer Institute and Hospital, Key Laboratory of Breast Cancer Prevention and Therapy, Tianjin Medical University, Ministry of Education, Tianjin 300060, China; ^2^Department of Pathology, Tianjin Medical University Cancer Institute and Hospital, National Clinical Research Center of Cancer, Tianjin 300060, China

**Keywords:** Breast cancer, positive lymph nodes, postmastectomy radiotherapy (PMRT), locoregional failure-free survival (LRFFS), overall survival (OS)

## Abstract

**Objective:**

To explore the effects of postmastectomy radiotherapy (PMRT) on the locoregional failure-free survival (LRFFS) and overall survival (OS) of breast cancer patients under different tumor stages and with one to three positive axillary lymph nodes (ALNs).

**Methods:**

We conducted a retrospective review of 527 patients with one to three positive lymph nodes who underwent modified radical or partial mastectomy and axillary dissection from January 2000 to December 2002. The patients were divided into the T_1_-T_2_ N_1_ and T_3_-T_4_ N_1_ groups. The effects of PMRT on the LRFFS and OS of these two patient groups were analyzed using SPSS 19.0, Pearson’s χ^2^-test, Kaplan-Meier method, and Cox proportional hazard model.

**Results:**

For T_1_-T_2_ N_1_ patients, no statistical significance was observed in the effects of PMRT on LRFFS [hazard ratio (HR)=0.726; 95% confidence interval (CI): 0.233-2.265; *P*=0.582] and OS (HR=0.914; 95% CI: 0.478-1.745; *P*=0.784) of the general patients. Extracapsular extension (ECE) and high histological grade were the risk factors for LRFFS and OS with statistical significance in multivariate analysis. Stratification analysis showed that PMRT statistically improved the clinical outcomes in high-risk patients [ECE (+), LRFFS: *P*=0.026, OS: *P*=0.007; histological grade III, LRFFS: *P*<0.001, OS: *P*=0.007] but not in low-risk patients [ECE (–), LRFFS: *P*=0.987, OS: *P*=0.502; histological grade I-II, LRFFS: *P*=0.816, OS: *P*=0.296]. For T_3_-T_4_ N_1_ patients, PMRT effectively improved the local control (HR=0.089; 95% CI: 0.210-0.378; *P*=0.001) of the general patients, whereas no statistical effect was observed on OS (HR=1.251; 95% CI: 0.597-2.622; *P*=0.552). Absence of estrogen receptors and progesterone receptors (ER/PR) (–) was an independent risk factor. Further stratification analysis indicated a statistical difference in LRFFS and OS between the high-risk patients with ER/PR (–) receiving PMRT and not receiving PMRT [ER/PR (–), LRFFS: *P*=0.046, OS: *P*=0.039]. However, PMRT had a beneficial effect on the reduction of locoregional recurrence (LRR) but not in total mortality [ER/PR (+), LRFFS: *P*<0.001, OS: *P*= 0.695] in T_3_-T_4_ N_1_ patients with ER/PR (+) who received endocrine therapy.

**Conclusion:**

PMRT could reduce ECE (+), histological grade III-related LRR, and total mortality of T_1_-T_2_ N_1_ patients. T_3_-T_4_ N_1_ patients with ER/PR (–) could benefit from PMRT by improving LRFFS and OS. However, PMRT could only reduce LRR but failed to improve OS for T_3_-T_4_ N_1_ patients with ER/PR (+) who received endocrine therapy.

## Introduction

Postmastectomy radiotherapy (PMRT), as a treatment modality for postoperative patients with breast cancer, is primarily used to reduce locoregional recurrence (LRR) and improve survival, although modestly, in patients with high-risk factors^1^^-^^4^.

According to the National Comprehensive Cancer Network (NCCN) guidelines^5^, PMRT should be considered for patients with T_3_-T_4_ breast cancer with more than three positive lymph nodes or with T_1_-T_2_ breast cancer with one to three positive lymph nodes. Given that several clinical and pathological factors may affect prognosis of patients with intermediate-risk breast cancer, using T/N classification only is an imprecise method in determining whether a patient should be considered for PMRT^6^^-^^9^. Several researchers have attempted to identify the risk factors for LRR and mortality after mastectomy to select patients who are most likely to benefit from PMRT^1^^-^^4^^,^^6^^-^^18^. However, these patient subgroups have not been clearly defined, and the contribution of PMRT to locoregional control and survival remains unclear.

The function of PMRT is not clearly defined in breast cancer patients with one to three positive lymph nodes. In this retrospective study, we identified prognostic factors for LRR and mortality of T_1_-T_2_ N_1_ and T_3_-T_4_ N_1_ breast cancer patients. In addition, we compared the locoregional failure-free survival (LRFFS) and overall survival (OS) of the high-risk patients with and without PMRT to define a subgroup of patients who might benefit from PMRT.

## Materials and methods

### Clinical data

From January 2000 to December 2002, breast cancer patients with pathologically proven one to three positive axillary lymph nodes (ALNs) were treated with modified radical mastectomy plus axillary dissection at the Tianjin Cancer Hospital. Of the 527 patients with one to three positive lymph nodes, the median age was 48.73 years (range, 26 to 79 years). The median number of involved ALNs was 1.93 (range, 1 to 3). A total of 432 patients with T_1_-T_2_ disease and 95 patients with T_3_-T_4_ disease were included in the study, 75.7% (327/432) and 70.5% (67/95) of whom received PMRT, respectively. The study was approved by the institutional ethics committee.

### Systemic treatment

All patients received TEC-based (docetaxel, epirubicin, cyclophosphamide) or docetaxel-containing regimens as adjuvant chemotherapy. Adjuvant endocrine therapy was performed for 5 years in all patients who had positive hormone receptors. Among 527 patients, 74.8% (394/527) underwent PMRT, which was delivered to the breast, chest wall, internal mammary, supraclavicular, and axillary fossa drawing region by medial and lateral-tangential fields with external-beam irradiation (4 or 6 MV photons/60 Co). The standard dose to the entire chest wall was 50 Gy (range, 46 to 54 Gy), 1.8 to 2 Gy/d, and five times weekly. The supraclavicular region and the full axilla were treated with a dose of 50 Gy using an anterior field. An additional external boost with electrons (2 Gy/10 Gy to 14 Gy) was performed in patients who had locally advanced disease.

### Follow-up

The median time of follow-up was 127.82 months (range, 15 to 155 months). All intervals were calculated from the date of completion of surgery, and the endpoint was defined as the last follow-up or death. Evaluation of tumor control was performed for patients in 4-month intervals for the first 2 years and in 6-month intervals for the next 3 years. Subsequently, these patients were observed on a yearly basis. Clinical examinations, which included blood sampling, routine chest radiograph, mammograph, and ultrasound, were performed as evaluation during the follow-up. Further evaluations were conducted only if the clinical findings indicated a disease progression. Survival period was calculated from the date of surgical resection to the date of last follow-up. The endpoints of interest included LRFFS and OS.

### Recurrence

LRR was identified as local recurrence (chest wall alone) or regional recurrence (axillary, supraclavicular, and internal mammary lymph nodes alone). Any recurrence outside these areas was defined as distant metastasis (DM).

### Statistical analysis

All analyses were performed using SPSS 19.0. Pearson’s χ^2^-test was used to compare the proportions of categorical covariates among the groups of patients with different T stages. OS and LRFFS were analyzed with Kaplan-Meier method. Univariate and multivariate hazard ratios (HR) and their 95% confidence intervals (CIs) were calculated using Cox’s proportional hazard model. A probability level of ≤0.05 was considered statistically significant.

## Results

### Basic information

**Figure 1:**
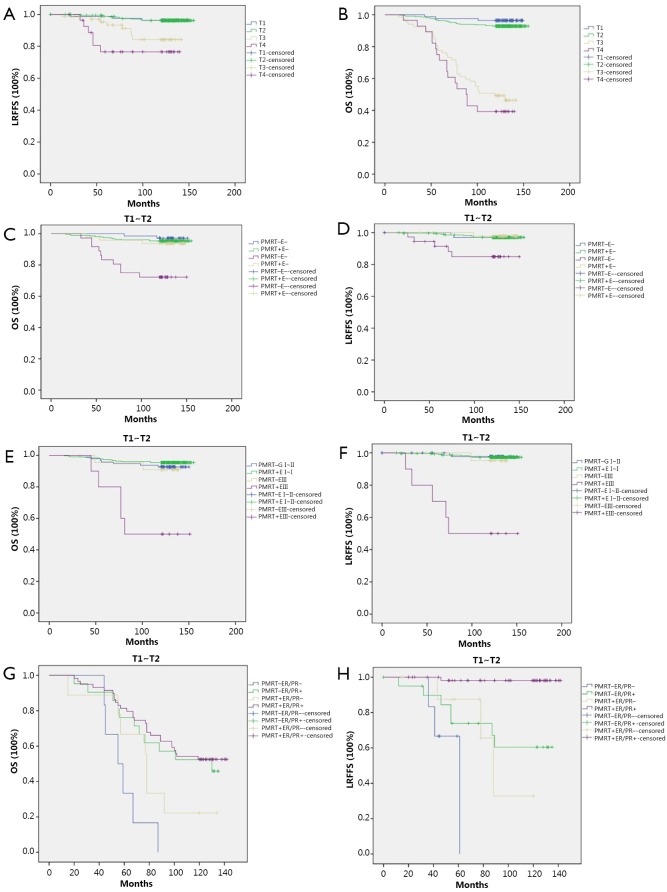
(A) Kaplan-Meier curve of LRFFS in different T stages; (B) Kaplan-Meier curve of OS in different T stages; (C) Kaplan-Meier curve of OS in patients with different ECE in T_1_-T_2_ N_1_ patients. PMRT+E– *vs*. PMRT–E–: *P*=0.502; PMRT+E+ *vs*. PMRT–E+: *P*=0.007 (PMRT–, non-PMRT; PMRT+, PMRT; E–, ECE–; E+, ECE+); (D) Kaplan-Meier curve of LRFFS in patients with different ECE in T_1_-T_2_ N_1_ patients. PMRT+E– *vs*. PMRT–E–: *P*=0.987; PMRT+E+ *vs*. PMRT-E+: *P*=0.026; (E) Kaplan-Meier curve of OS in patients with different histological grades in T_1_-T_2_ N_1_ patients. PMRT+ GI-II *vs*. PMRT– GI-II: *P*=0.296; PMRT– GIII *vs*. PMRT+ GIII: *P*=0.007. (GI-II, grade I-II; GIII, grade III); (F) Kaplan-Meier curve of LRFFS in patients with different histological grades in T_1_-T_2_ N_1_ patients. PMRT+ GI-II *vs*. PMRT– GI-II: *P*=0.816; PMRT– GIII *vs*. PMRT+ GIII: *P*<0.001; (G) Kaplan-Meier curve of OS in patients with different hormone receptor status in T_3_-T_4_ N_1_ patients. PMRT+ER/PR– *vs*. PMRT–ER/PR–: *P*=0.039; PMRT+ER/PR+ *vs*. PMRT–ER/PR+: *P*=0.695; (H) Kaplan-Meier curve of LRFFS in patients with different hormone receptor status in T_3_-T_4_ N_1_ patients. PMRT+ER/PR– *vs*. PMRT–ER/PR–: *P*=0.046; PMRT+ER/PR+ *vs*. PMRT–ER/PR+: *P*<0.001.

With a median follow up of 127.82 months (range, 15 to 155 months), 3.7% (16/432) and 14.7% (14/95) of patients developed LRR in T_1_-T_2_ N_1_ and T_3_-T_4_ N_1_ patient groups, respectively. OS was 93.5% (404/432) and 45.3% (43/95) in the T_1_-T_2_ N_1_ and T_3_-T_4_ N_1_ groups, respectively. The Kaplan-Meier curves of LRFFS and OS in different T stages confirmed the statistically significant difference in LRFFS and OS between the T_1_-T_2_ N_1_ and T_3_-T_4_ N_1_ patients (**Figure 1A,B**). The distribution patterns of clinico-pathologic characteristics for the PMRT and non-PMRT groups are presented in **Table 1**. A statistically significant difference was observed between the two groups regarding the status of extracapsular extension (ECE) and the number of involved ALNs (*P*<0.05).

**Table 1:** Clinicopathologic features of patients in the study

Characteristics	No. of patients		*P*
	PMRT	No-PMRT	
Age, yrs			0.841
≤50	208	72	
>50	186	61	
ER/PR			0.052
Negative	54	28	
Positive	340	105	
Postmenopausal			0.133
No	201	78	
Yes	193	55	
ECE			<0.001
Negative	324	87	
Positive	70	46	
Histological grade			0.21
I-II	364	118	
III	30	15	
T stage			0.299
T_1_-T_2_	327	105	
T_3_-T_4_	67	28	
Endocrine therapy			0.131
No	57	27	
Yes	337	106	
ALN			<0.001
1	158	38	
2	129	25	
3	107	70	

ER, estrogen receptor; PR, progesterone; ER/PR, estrogen receptor and progesterone receptor; ECE, extracapsular extension; ALN, axillary lymph nodes; PMRT, postmastectomy radiotherapy.

### Univariate and multivariate analyses

The univariate and multivariate factors for LRR in the different T stages were analyzed using Cox’s proportional hazard model. The risk and protective factors in T_1_-T_2_ and T_3_-T_4_ patients were different. ECE (HR=2.867; 95% CI: 1.035-7.939; *P*=0.043) and histological grade III (HR=9.219; 95% CI: 2.956-28.747; *P*=0.000) were the risk factors in T_1_-T_2_ patients. For the T_3_-T_4_ patients, the risk factor was estrogen receptor and progesterone receptor (ER/PR) (–) tumors, whereas the protective factors were ER/PR (+) tumors (HR=0.098; 95% CI: 0.025-0.389; *P*=0.001) and PMRT (HR=0.089; 95% CI: 0.210-0.378; *P*=0.001) (**Tables 2****,****3**).

**Table 2:** Multivariate analysis with Cox proportional hazards model for OS and LRFFS of T_1_-T_2_ N_1_ patients

Variable	OS				LRFFS		
	HR	95% CI	*P*		HR	95% CI	*P*
Histological grade	3.365	1.332-8.602	0.010		9.219	2.956-28.747	0.000
ER/PR	1.716	0.679-4.333	0.627		1.375	0.390-4.849	0.362
PMRT	0.914	0.478-1.745	0.784		0.726	0.233-2.265	0.582
ECE	1.086	1.012-1.164	0.022		2.867	1.035-7.939	0.043

OS, overall survival; LRFFS, locoregional failure-free survival; HR, hazard ratio; CI, confidence interval; ER/PR, estrogen receptor and progesterone receptor; PMRT, postmastectomy radiotherapy; ECE, extracapsular extension.

**Table 3:** Multivariate analysis with cox proportional hazards model for OS and LRFFS of T_3_-T_4_ N_1_ patients

Variable	OS				LRFFS		
	HR	95% CI	*P*		HR	95% CI	*P*
Histological grade	1.845	0.957-3.556	0.067		0.337	0.063-1.795	0.202
ER/PR	0.307	0.154-0.610	0.001		0.098	0.025-0.389	0.001
PMRT	1.251	0.597-2.622	0.552		0.089	0.210-0.378	0.001
ECE	0.979	0.533-1.801	0.947		2.702	0.633-11.529	0.179

OS, overall survival; LRFFS, locoregional failure-free survival; HR, hazard ratio; CI, confidence interval; PMRT, postmastectomy radiotherapy; ECE, extracapsular extension.

The factors affecting OS varied between the T_1_-T_2_ N_1_ and T_3_-T_4_ N_1_ patients. ECE (HR=1.086; 95% CI: 1.012-1.164; *P*=0.022) and histological grade III (HR=3.365; 95% CI: 1.332-8.602; *P*=0.010) were the risk factors in T_1_-T_2_ patients. However, the risk factor in T_3_-T_4_ patients was ER/PR (–) tumors. ER/PR (+) tumors (HR=0.307; 95% CI: 0.154-0.610; *P*=0.001) had a significant effect in improving OS (**Tables 2**** and ****3**).

### Effects of PMRT on LRFFS and OS of T_1_-T_2_ N_1_ patients based on ECE status and histological grade

The OS and LRFFS were analyzed by Kaplan-Meier, and survival curves were plotted for the T_1_-T_2_ N_1_ patient subgroups: ECE (–) /PMRT (–), ECE (–) /PMRT (+), ECE (+) /PMRT (–), and ECE (+) /PMRT (+). The log-rank test results showed that PMRT had statistically positive effects on improving LRFFS (*P*=0.026) and OS (*P*=0.007) of T_1_-T_2_ N_1_ patients with ECE (+) but not ECE (–). We also performed a subgroup analysis according to the histological grade, and the results showed that PMRT could improve the LRFFS (*P*<0.001) and OS (*P*=0.007) of T_1_-T_2_ N_1_ patients with histological grade III (**Figure 1C-F**).

### Effects of PMRT on LRFFS and OS of T_3_-T_4_ N_1_ patients based on hormone receptor status

With regard to LRFFS and OS of T_3_-T_4_ N_1_ patients, ER/PR (+) was a statistically significant factor on multivariate analysis. PMRT was beneficial on LRFFS of all patients regardless of the hormone receptor status. The effects of PMRT on LRFFS and OS of the patients with different ER/PR statuses were examined. All T_3_-T_4_ N_1_ patients were first stratified into subgroups of ER/PR (+) and ER/PR (–). We observed that PMRT was useful for the reduction of LRR (*P*<0.001) of T_3_-T_4_ N_1_ patients with ER/PR (+) but failed to improve OS (*P*=0.695). However, patients with ER/PR (–) could benefit from PMRT on improving LRFFS (*P*=0.046) and OS (*P*=0.039) (**Figure 1G,H**).

## Discussion

The significance of PMRT to reduce LRR and total mortality in the subgroup of patients with one to three positive lymph nodes remains unclear^7^^,^^11^^-^^16^. Currently, the indication of PMRT is mainly determined by the number of positive lymph nodes and the T stage. However, some studies^10^^,^^16^^,^^19^^,^^20^ have reported the comparatively more effective prognostic predictors other than T and N stage that guide the PMRT treatment. These predictors include age, hormone receptor status, ECE status, histological grade, lymphovascular invasion, menstrual status, and lymph node ratio.

Huang *et al*.^12^ highly recommends the PMRT to breast cancer patients with T_1_-T_2_ and one to three positive lymph nodes for reducing LRR and improving disease-free survival. Tendulkar *et al*.^16^ suggested that PMRT provides excellent locoregional control for patients with one to three positive lymph nodes, regardless of PMRT patients in more advanced stage (about 40% had stage T_3_-T_4_ disease) and a greater number of risk factors, such as pathological grade III and ECE. However, Geng *et al*.^17^ suggested that PMRT does not significantly improve the LRFFS for patients with one to three positive axillary nodes, regardless of the ECE status. Kong *et al*.^18^ found that PMRT does not improve LRR, DM-free survival, or OS in T_1_-T_2_ N_1_ breast cancer patients. However, PMRT might be beneficial in a subgroup of patients with histological grade III disease, ECE, or triple-negative subtype. PMRT is important in identifying the risk factors associated with increased risk of LRR and total mortality in patients with one to three positive axillary lymph nodes to establish its indications.

According to the American Society of Clinical Oncology^21^, insufficient evidence exists to formulate recommendations or suggestions for the routine use of PMRT in patients with T_1_-T_2_ breast cancer and one to three positive lymph nodes. However, PMRT has been considered for T_1_-T_2_ N_1_ patients based on the NCCN guidelines^5^. Our retrospective study provided some new information with regard to patients with one to three positive axillary lymph nodes, who may benefit from PMRT.

Based on our study, different effects of PMRT on improving LRFFS or OS were found between the T_1_-T_2_ N_1_ and T_3_-T_4_ N_1_ patients. Previous studies have reported^15^^,^^16^ that the LRFFS and OS of T_1_-T_2_ N_1_ breast cancer patients treated with radical mastectomy are dependent on several prognostic factors other than T and N stage. Our analysis revealed that ECE (+) and histological grade III were the high-risk factors for LRR and mortality of T_1_-T_2_ N_1_ patients. The stratification analysis results revealed that PMRT had a positive effect in reducing ECE (+) or histological grade III-related LRR and mortality. However, the remaining patients with ECE (–) or histological grade I-II experienced extremely low LRR and mortality rates after mastectomy treatment, and the benefit from PMRT was minimal. Although PMRT had no protective function in improving LRFFS and OS of the general T_1_-T_2_ N_1_ patients, high-risk patients with ECE (+) and histological grade III could benefit from PMRT.

Contrary to T_1_-T_2_ N_1_ patients, the general T_3_-T_4_ N_1_ patients could benefit from PMRT in terms of LRFFS but not in OS. Stage T_3_-T_4_ is a high-risk factor in breast cancer patients, who are more likely to develop DM than patients with early T stage disease. Breast cancer tends to be a systemic disease with potential sub-clinical DM in Fisher’s theory^17^. Our analysis revealed that PMRT could improve the LRR control in T_3_-T_4_ patients, but no statistically significant effect on OS was observed among these patients. In addition, patients with ER/PR (+) benefited from endocrine therapy. All patients with ER/PR (+) who were included in our study received endocrine therapy. Endocrine therapy was a protective factor to improve LRFFS and OS of T_3_-T_4_ N_1_ patients according to the multivariate analysis results. Thus, the risks of LRR and mortality were positively associated with ER/PR (–). NCCN guidelines^5^ suggested that T_3_-T_4_ patients should receive PMRT. Rangan *et al*.^22^ reported that LRR rate of patients with one to three positive lymph nodes who received chemotherapy and endocrine therapy is approximately 10% under the condition of non-PMRT. To further determine whether PMRT is essential for patients receiving endocrine therapy and whether ER/PR (–) patients could benefit from it, we analyzed its effects on LRFFS and OS of T_3_-T_4_ N_1_ patients with ER/PR (–) and who received endocrine therapy, respectively. The results of stratification analysis indicated that PMRT caused a statistically significant improvement in LRFFS and OS of T_3_-T_4_ N_1_ patients with ER/PR (–). For T_3_-T_4_ N_1_ patients who received endocrine therapy, PMRT could improve local control but no statistical change in OS was observed compared with non-PMRT.

PMRT alleviates local symptoms but often results in significant pathological damage to the heart, lungs, and skin. A meta-analysis by Taghian *et al*.^19^ revealed a significant increase in non-breast cancer mortality in irradiated women. The mortality is mainly because of heart disease and lung cancer. Given the complications of PMRT, its necessity for T_3_-T_4_ N_1_ patients receiving endocrine therapy should be reconsidered because no statistical effect on OS was observed in this study despite the improvement in local control.

## Conclusion

According to our results, PMRT is highly recommended to improve LRFFS and OS for T_1_-T_2_ N_1_ patients with ECE (+) or pathological grade III as well as for T_3_-T_4_ N_1_ patients with ER/PR (–). However, PMRT has to be reconsidered for T_3_-T_4_ N_1_ patients with ER/PR (+) who benefited from endocrine therapy on improving LRFFS and OS. Other prognostic factors should be considered, and the decision has to be made individually on the basis of endocrine therapy and request of the patient because PMRT could control LRR but not total mortality.
